# Interleukin-5: an indicator of mild cognitive impairment in patients with type 2 diabetes mellitus - a comprehensive investigation ranging from bioinformatics analysis to clinical research

**DOI:** 10.1007/s40618-024-02430-2

**Published:** 2024-09-30

**Authors:** Hui Zhang, Wenwen Zhu, Shufang Yang, Tong Niu, Huzaifa Fareeduddin Mohammed Farooqui, Bing Song, Hongxiao Wang, Sumei Li, Jumei Wang, Linlin Xu, Zhen Zhang, Haoqiang Zhang

**Affiliations:** 1 Henan Key Laboratory of Rare Diseases, Endocrinology and Metabolism Center, The First Affiliated Hospital, College of Clinical Medicine of Henan, University of Science and Technology, Luoyang, China; 2 Department of Endocrinology, Affiliated Zhongda Hospital of Southeast University, Nanjing, China; 3 Department of Endocrinology, Taizhou People’s Hospital, Taizhou, China; 4 Department of Endocrinology, The First Affiliated Hospital of Jinzhou Medical University, Jinzhou, China; 5 Department of Endocrinology, Institute of Endocrine and Metabolic Diseases, Centre for Leading Medicine and Advanced Technologies of IHM, The First Affiliated Hospital of USTC, Division of Life Sciences and Medicine, University of Science and Technology of China, Hefei, 230001 China

**Keywords:** Type 2 diabetes mellitus, Mild cognitive impairment, Neuroinflammation, Interleukin-5

## Abstract

**Purpose:**

Neuroinflammation constitutes an underlying mechanism for cognitive impairment. Here, we endeavor to scrutinize the potential contribution of interleukin-5 (IL-5) towards mild cognitive impairment (MCI), and to assess its diagnostic value for MCI in patients with type 2 diabetes mellitus (T2DM).

**Methods:**

RNA-seq was used to explore the potential neuroinflammation factors in the hippocampus of diabetic mice with cognitive decline. Additionally, the promising risk factor was verified in animals. Finally, the association between IL-5 levels and cognitive function and its diagnostic value for MCI were assessed.

**Results:**

In animals, up-regulated IL-5 mRNA and protein levels were detected by RNA-seq and (or) verified experiments in the hippocampus of diabetic db/db mice with cognitive decline, compared to those of db/m mice without diabetes. In human, compared to diabetic patients without MCI, those with MCI demonstrate elevated levels of IL-5. It is natively associated with Montreal Cognitive Assessment (MoCA) scores, reflecting global cognitive function, and positively correlated with Trail Making Test A (TMTA) scores, reflecting information processing speed. Furthermore, an elevated level of IL-5 is identified as a risk factor for MCI, and a factor that influences TMTA scores. Finally, it is recommended that the cut-off value for IL-5 in the diagnosis of MCI is 22.98 pg/mL, with a sensitivity of 68.6% and specificity of 72.9%.

**Conclusions:**

IL-5 is considered a risk factor for MCI in T2DM patients and is associated with their performance in information processing speed. Moreover, an elevated level of IL-5 is a plausible biomarker for MCI in T2DM patients.

## Introduction

In 2021, a total of 537 million individuals across the globe were diagnosed with diabetes. According to estimates, this number is expected to increase to 643 million and 783 million by 2030 and 2045, respectively [1]. Among the various types of diabetes, T2DM is the most prevalent, and is characterized by persistent hyperglycemia, insulin resistance, oxidative stressand chronic inflammation [2], which are associated with a range of diabetic complications, including neuronal dysfunctions [3–8]. MCI represents an intermediary stage between normal cognition and dementia [9], and its early identification and management could potentially prevent disease progression and even restore normal cognitive function [10]. Recently, our study demonstrated that elevated levels of inflammation factors are linked to MCI, particularly memory deficits, in patients with T2DM [11, 12].

Our recent studies suggest that cholesterol [13, 14] and fatty acid [15] disorders are associated with cognitive impairment in patients with T2DM. IL-5 is a cytokine associated with inflammation and is released from group II innate lymphoid cells. It is closely related to allergic inflammation, parasitic infections, and even lipid metabolism [16]. Indeed, IL-5 is also associated with cognitive function in both children [17] and adults [18], irrespective of diabetes status. Furthermore, studies suggest that IL-5 is associated with cognitive function in animals as well [19, 20].

Regarding T2DM, serum levels of IL-5 are significantly higher in patients with T2DM than in the control group [21]. Patients with non-proliferative diabetic retinopathy and proliferative diabetic retinopathy exhibit elevated IL-5 levels compared to diabetic patients without retinopathy. Additionally, the concentration of IL-5 is higher in the proliferative diabetic retinopathy group than in the non-proliferative diabetic retinopathy group [22]. IL-5 mRNA levels are also increased in patients with diabetic nephropathy compared to those without diabetic nephropathy [23]. These studies suggest that IL-5 may not only be related to the occurrence of diabetes but also to the development of diabetic complications. Our previous studies have suggested that levels of brain-derived neurotrophic factor in plasma are associated with memory prognosis in long-term T2DM patients [24]. Interestingly, down-regulated levels of brain-derived neurotrophic factor were observed in functional dyspepsia patients, while elevated levels of IL-5 were detected in those patients [25]. Additionally, IL-5 is a member of the Interleukin-3 (IL-3) family. Upon binding with its receptor, IL-5, along with other members of the IL-3 family, can potentially participate in neuronal apoptosis and subsequently influence cognitive function through the JAK/STAT signaling pathway. This may be one of the mechanisms of diabetic dysfunction [26–30]. These findings suggest potential associations between diabetic cognitive impairment and IL-5 levels.

Although IL-5 is both associated with cognitive dysfunction and diabetes (including diabetic complications), the relationship between IL-5 and MCI in T2DM patients is still unclear. Generally, there may be a potential correlation between IL-5 and MCI in patients with T2DM. However, the specific nature of this relationship requires further confirmation. Moreover, the connection between IL-5 and more intricate cognitive functions remains to be explored. Early detection of MCI can be beneficial, but its latent onset and mild symptoms present challenges in timely recognition. Presently, diagnosis of MCI relies on evaluation of relevant scales, which can be subjective and time-consuming. Furthermore, invasive examinations may be needed, and imaging studies can be prohibitively expensive. Consequently, there is an urgent need for minimally invasive diagnostic markers that are easy to perform. As such, IL-5 has the potential to serve as a biomarker that can be easily detected in peripheral blood. Thus, our study aims to investigate the association between MCI (including cognitive performance details) and IL-5 levels in patients with T2DM. Additionally, we evaluate the diagnosis cut-off point and diagnosis value by using the ROC curve.

## Materials and methods

### Animal housing

The investigation encompassed the utilization of 7-week-old male db/db mice (*n* = 10) and db/m mice (*n* = 10), obtained from Beijing HFK Biotechnology Co., Ltd. (Beijing, China), and subsequently housed in a specific pathogen-free animal facility. After a duration of 16 weeks, all experimental mice underwent anesthesia through the administration of 4% halothane, followed by humane euthanasia via cervical vertebra dislocation. Post-euthanasia, both blood and brain tissue specimens were expeditiously collected and preserved at -80 °C to facilitate subsequent research endeavors. It is noteworthy that the research was conducted in strict accordance with the principles delineated in the “Guide for the Care and Use of Laboratory Animals” and received ethical clearance from the Animal Studies Committee at our institution.

### Morris water maze

The Morris water maze paradigm was employed for the assessment of cognitive function subsequent to the dietary interventions elucidated earlier, in adherence to established methodologies articulated in previous investigations [31]. During the initial test session, mice underwent a one-minute period of unimpeded swimming to acclimate themselves to the environment, devoid of the presence of the concealed platform. Subsequent to this acclimatization phase, each mouse participated in a five-day training regimen within the maze, wherein a concealed platform was strategically positioned within the apparatus. Quantitative parameters, specifically the escape latency (defined as the time taken by each mouse to locate the concealed platform) and the path length traversed by each mouse, were systematically documented throughout the training period. On the sixth day of testing, supplementary performance metrics were recorded in the absence of the platform, encompassing the percentage of time spent in the target quadrant, the frequency of crossings over the platform area, and the swimming speed of the mice.

### mRNA library construction, sequencing and functional enrichment analysis

The information for mRNA library construction and sequencing are shown in Supplementary File 1. All sequencing procedure were performed by Shenyang Jianji Biotechnology Co., Ltd.

### Real time polymerase chain reaction (RT-PCR)

Total RNA was extracted using TRIzol reagent (Thermo Fisher, USA, Catalogue No.: 15,596,026). Subsequently, the isolated RNA underwent reverse transcription into cDNA employing HiScript III RT SuperMix (Vazyme, Nanjing, China, Catalogue No.: R223-01), following the manufacturer’s provided protocols. Quantitative real-time PCR was conducted on the Applied Biosystems StepOnePlus system utilizing SYBR Green (Vazyme, Nanjing, China, Catalogue No.: Q341-02). The primer sequences employed were as follows: IL-5 forward primer, 5′-ATGGAGATTCCCATGAGCAC-3′ and reverse primer, 5′-GTCTCTCCTCGCCACACTTC-3′; GAPDH forward primer, 5′-CGGAGTCAACGGATTTGGTCGTAT-3′ and reverse primer, 5′-AGCCTTCTCCATGGTGGTGAAGAC-3′. The PCR cycling conditions comprised an initial denaturation step (95 °C for 2 min) followed by 40 cycles of denaturation (95 °C for 15s), annealing (55 °C for 30s), extension (72 °C for 30s), and acquisition temperature (15s). Gene expression analysis was conducted using the 2^−(ΔCt)^ method, normalizing to GAPDH as the reference gene.

### Western blotting (WB)

The Western blotting procedure was conducted in accordance with a previously established protocol [32] outlined as follows. Hippocampal tissue was isolated for protein extraction utilizing radioimmunoprecipitation (Wanlei Biotechnology Co. Ltd, Shenyang, China, Catalogue No.: WLA016a) and quantified using a BCA assay following the manufacturer’s instructions (Wanlei Biotechnology Co. Ltd, Shenyang, China, Catalogue No.: WLA004b). Subsequently, proteins were resolved on SDS-PAGE gels and transferred onto polyvinylidene fluoride membranes. Primary antibodies, specifically rabbit anti-mouse antibodies, were employed to selectively bind target proteins such as IL-5 (Wanlei Biotechnology Co. Ltd, Shenyang, China, Catalogue No.: WLA 01401) and β-actin (Wanlei Biotechnology Co. Ltd, Shenyang, China, Catalogue No.: WLA01372) at 4 °C overnight. Following primary antibody binding, incubation with goat-anti-rabbit secondary antibodies conjugated with HRP (Wanlei Biotechnology Co. Ltd, Shenyang, China, Catalogue No.: WLA023a) was conducted. Detection of protein levels was accomplished through the utilization of an ECL kit (Wanlei Biotechnology Co. Ltd, Shenyang, China, Catalogue No.: WLA006a) prior to exposure.

### Immunofluorescence

Fresh mouse brains were harvested and subjected to embedding in optimal cutting temperature compound (OTC) for the preparation of frozen Sect. (40 μm) inclusive of the hippocampus. Prior to overnight incubation at 4 °C with rabbit anti-mouse primary antibody to IBA-1 (Huabio, Hangzhou, China, Catalogue No.: ET1705-78) and rat anti-mouse primary antibody CD68 (Bio-Rad, Oxford, UK, Catalogue No.: MCA1957T), all sections underwent blocking in phosphate-buffered saline (PBS) containing 5% normal goat serum for 1 h at room temperature. Following triple washes with PBS, detection of primary antibodies was achieved using FITC-conjugated goat anti-rabbit secondary antibody (Servicebio, Wuhan, China, Catalogue No.: GB22302) and Cy3-conjugated goat anti-rat secondary antibody (Servicebio, Wuhan, China, Catalogue No.: GDP1012) in a light-protected environment. Subsequent to three additional PBS washes, visualization of IBA-1 and CD68 was conducted utilizing a confocal microscope.

### Enzyme-linked immunosorbent assay (ELISA)

In conjunction with the aforementioned measurements, blood samples were procured simultaneously and processed to obtain plasma. Specifically, collected blood samples were subjected to centrifugation at 4 °C for 30 min, with a relative centrifugal force of 1000 g. The resulting plasma samples were then subjected to quantification of IL-5 levels by the kit of enzyme-linked immunosorbent assay following the manufacturer’s protocol for mouse (Cloud-Clone Corp., China, Wuhan, Catalogue No.: SEA078Mu) and human (Cloud-Clone Corp., China, Wuhan, Catalogue No.: SEA078Hu). Additionally, levels of TNF-ɑ, IL-6, IL-1β, and IL-10 were measured in the protein extraction solution from hippocampal tissue of mice by similar kits (Cloud-Clone Corp., China, Wuhan, Catalogue No.: MEA133Mu, SEA079Mu, SEA563Mu, and SEA056Mu).

### Clinical study design and clinical ethical approval

A total of 105 patients were recruited from Department of Endocrinology at Taizhou People’s Hospital, all of whom met the diagnostic criteria for T2DM. Out of these patients, 35 were identified as having MCI and were subsequently allocated to the MCI group. The remaining 70 patients who exhibited normal cognitive function were allocated to the control group. Prior to participation in the study, all participants were provided with information regarding the purpose and procedures of the investigation, and subsequently provided informed consent by signing their name. This study was ethically approved by the Ethics Committee for Medical Research at Taizhou People’s Hospital (Approval No.: 202,204,601). All study participants met the World Health Organization’s 1999 Criteria for diabetes [33] and had a diabetes duration of over three years. Of the recruited participants, 35 met the criteria for MCI according to the MCI Working Group of the European Consortium on Alzheimer’s disease [34]. The remaining 70 participants did not meet the criteria for MCI but were included in the control group as they satisfied the standards for T2DM. The exclusion criteria were consistent with those used in a previous study [35] and specific described as bellow. (a) Recent diagnosed acute complications of diabetes; (b) severe low plasma glucose; (c) acute vascular disease of heart and brain; (d) alcohol or drug abuse; (e) diagnosed disease of thyroid (with thyroid dysfunction or abnormal autoimmune antibodies); (f) severe infection, major surgery, (g) visual or hearing dysfunction (cannot finish neuropsychological tests); (h) depression and dementia (severe cognitive decline out of the range of MCI); (i) smoking and drinking; (j) other diseases may affect (or potentially influence) cognition; cognitive function testing and inflammation, like thyroid function disorder, anemia, cancer, and autoimmune disease (e.g., Crohn’s disease, rheumatoid arthritis, systemic lupus erythematosus, and so on).

### Clinical data collection

The study collected clinical data from patients, including age, gender, duration of education and diabetes. Anthropometric measurements, such as weight and height, were taken upon the patients’ admission to the hospital, and body mass index (BMI) was calculated using the formula weight (kg)/height (m)^2^. Blood samples were collected on the second day of hospitalization to measure fasting plasma glucose (FPG), glycosylated hemoglobin (HbA1c), triglycerides (TG), total cholesterol (TC), high-density lipoprotein cholesterol (HDL-C), and low-density lipoprotein cholesterol (LDL-C) at the Center Laboratory of Taizhou People’s Hospital, for medical purposes. All data were obtained from the medical history of the patients. The measurement of IL-5 levels is described in detail above.

### Human neuropsychological tests

Human neuropsychological tests were performed according to our previous study [36]. The assessment of global cognitive abilities in this study was conducted using the MoCA scores. In cases where the education duration was less than 12 years, an additional score of 1 was added to the MoCA scores. The calculation of MoCA scores followed a previously reported method [37]. Information processing speed function was measured using the TMTA in accordance with a previous study [38]. The executive function was evaluated using the Digit Span Test (DST) [39], Verbal Fluency Test (VFT) [40] and Trail Making Test-B (TMTB) [38], based on previous studies. The function of scene memory was detected using the Logical Memory Test (LMT) [41].

### Human sample size calculation

The minimum sample size was calculated using PASS V11.0.7 (NCSS, USA). Upon completion of participants recruitment, the minimum sample size was estimated based on the ratio of patients in the MCI group to those in the control group, as well as the mean and standard deviation of IL-5 levels. Given a ratio of 1:2 between the MCI and control groups, the minimum sample sizes for the control and MCI groups were estimated to be 41 and 21 participants, respectively.

### Statistical methods

SPSS 22.0 (IBM, USA) was used for data analysis. Normally distributed variables in two groups were described as mean and standard deviation and compared Student’s t tests. Asymmetrically distributed variables in two groups were described as median and interquartile range and compared by nonparametric Mann–Whitney U tests. Binary variables were described as frequency and percentage. Their difference in two group was compared by chi-squared test. Pearson and partial correlation analyses was performed with or without adjust the factors (age and gender), respectively, in all patients and patients with MCI. Additionally, binary logistic analysis was employed to find the risk factors of MCI. Additionally, multiple linear regression was performed to explore the factors influence global cognitive function and information processing speed function. Finally, the diagnosis cut-off point and diagnosis values were also evaluated by from ROC curve (In this process, the highest J point is calculated by Youden’s index) [42].

## Results

### Compare of the cognitive performance between mice with or without diabetes

**Fig. 1:**
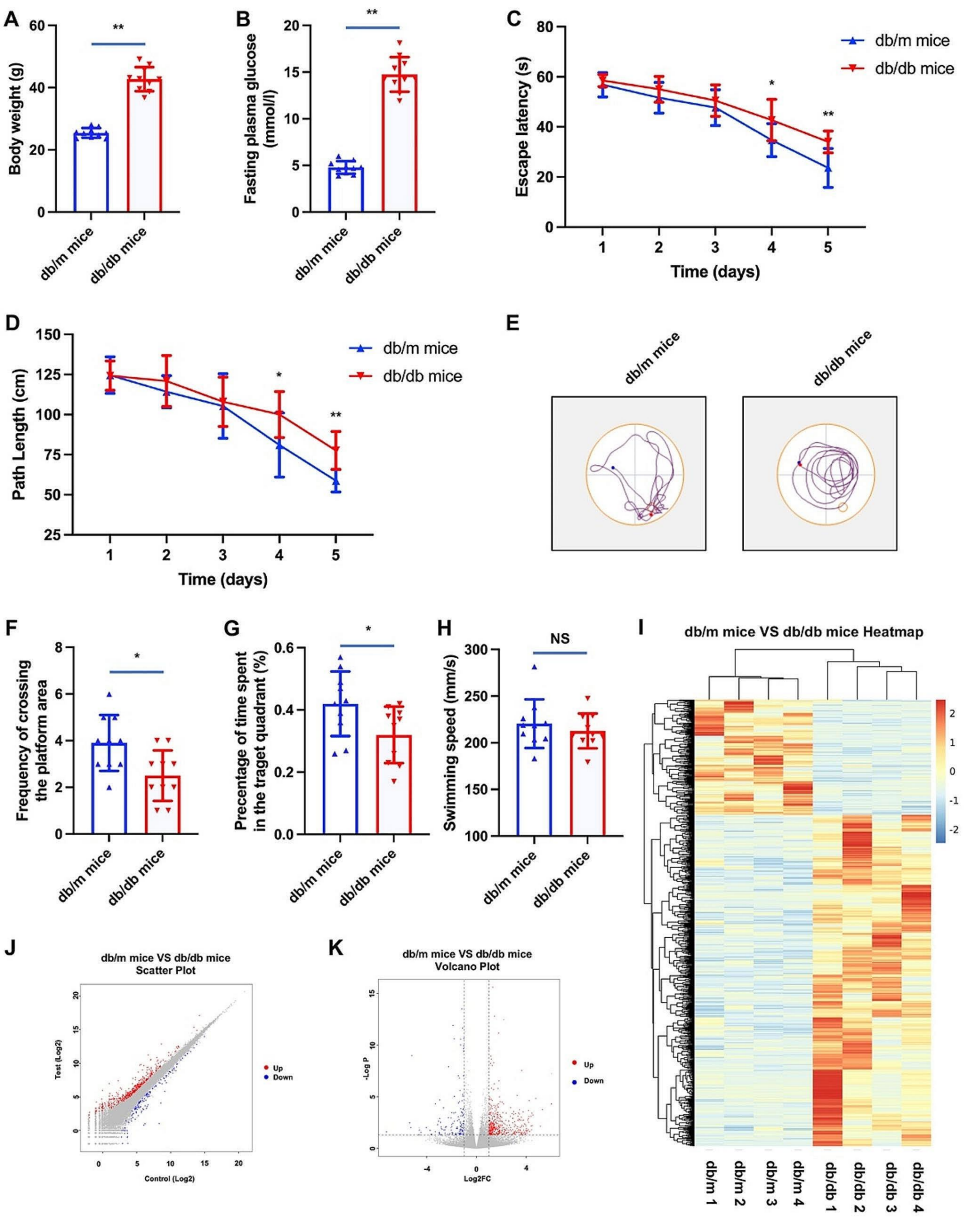
Cognitive performance and RNA-seq results of mice with and without diabetes. Notes for Fig. 1: ^*^P < 0.05, ^**^P < 0.01; Fig. 1A and B showed the difference of body weight and fasting plasma glucose of db/db mice and db/m mice. Figure 1 C and 1D showed the difference of escape latency and path length of db/db mice and db/m mice. Figure 1E showed the trajectory of movement in water maze of db/db mice and db/m mice. Figure 1 F and 1G showed the difference of frequency of crossing the platform area and percentage of the time spent in the target quadrant of db/db mice and db/m mice. Figure 1 H showed the difference of swimming speed of db/db mice and db/m mice. Figure 1I showed the heatmap of RNA-seq results of db/db mice and db/m mice. Figue 1 J and 1 K showed the scatter plot and volcano plot of db/db mice VS db/m mice

Hyperglycemia stands as a pivotal risk factor for cognitive dysfunction in both human subjects [43, 44] and animal models afflicted with diabetes mellitus [45, 46]. In pursuit of substantiating this assertion and elucidating potential underlying mechanisms, the present study employed db/db mice. Analogous to individuals with T2DM, db/db mice manifested heightened body weights and hyperglycemia (refer to Fig. 1A and B). Subsequent experimentation involving a water maze was conducted to assess cognitive performance. Notably, in comparison to their non-diabetic counterparts (db/m mice), db/db mice exhibited a significant decline in cognitive function. This was evidenced by notably shorter escape latencies and path lengths observed in the diabetic mice group (refer to Fig. 1C and D). Furthermore, discernible distinctions were observed in the reduced frequency of crossing the platform area (refer to Fig. 1E and F) and the percentage of time spent in the target quadrant (Fig. 1G) when comparing db/db mice to their non-diabetic counterparts. Despite an observable reduction in swimming velocity among diabetic mice, the incongruity relative to non-diabetic mice failed to achieve statistical significance (refer to Fig. 1H).

### Analysis of differentially expressed genes

To elucidate the differentially expressed genes (DEGs) between db/m mice without diabetes and ab/db mice with diabetes, a negative binomial distribution test was conducted to examine the difference in the number of reads. Genes meeting the criteria of an adjusted *P*-value < 0.05 and a fold change exceeding two were considered significant. The analysis revealed a total of 745 DEGs between db/m mice and db/db mice. Specifically, in comparison to db/m mice, 553 genes exhibited significant up-regulation, while 192 genes displayed significant down-regulation in db/db mice. The resulting gene expression profiles were visually represented through a cluster heatmap, a scatter plot, and a volcano plot (refer to Fig. 1I, J and K). The detailed catalog of the 745 DEGs can be found in Table file1.

### KEGG enrichment analysis

**Fig. 2:**
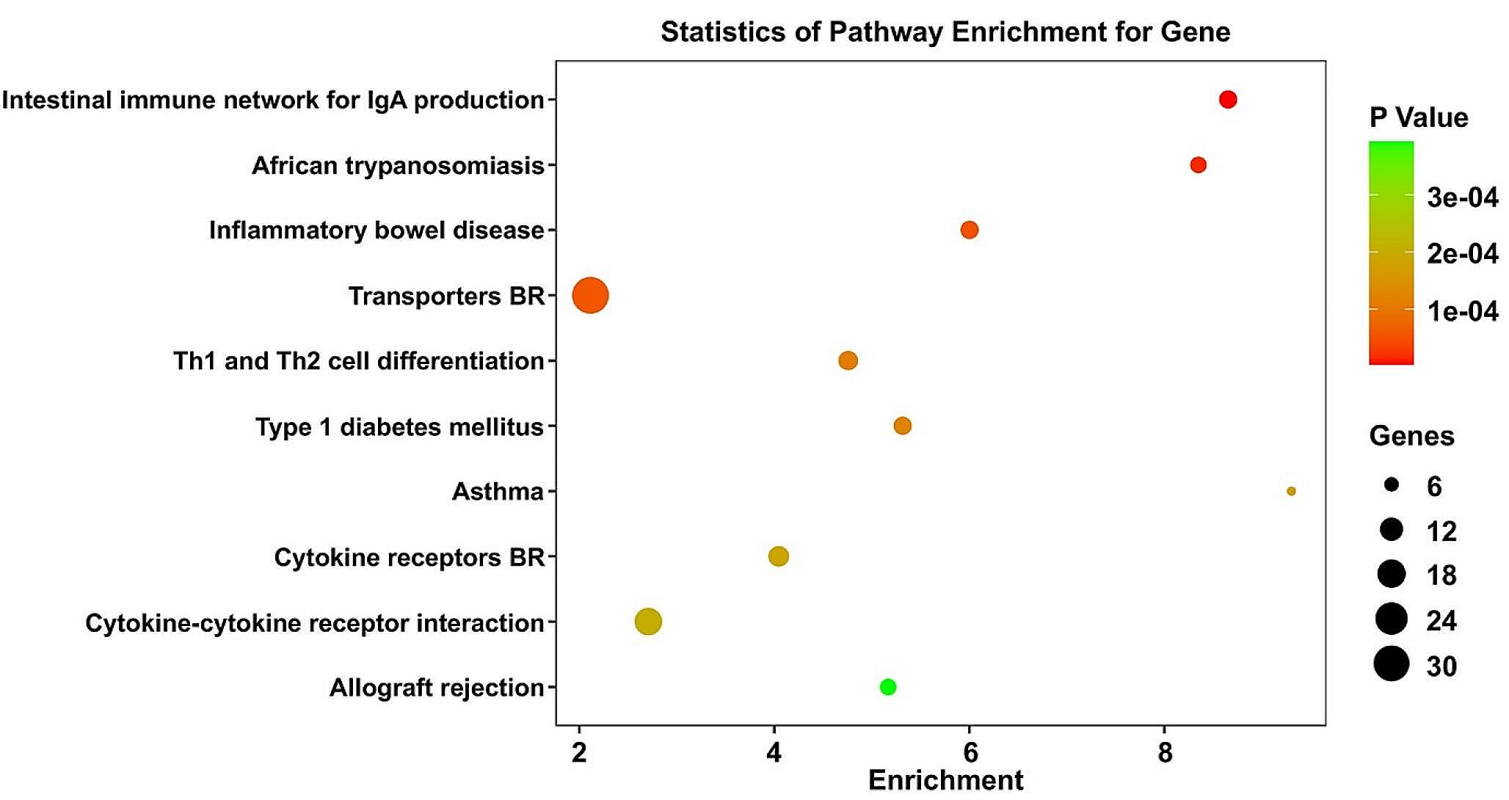
KEGG pathway analysis of DEGs in db/db mice and db/m mice. Notes for Fig. 2: The size of bubbles represents the number of genes in the pathway, while the color of bubbles represents *P*-value

The biological pathway analysis referred to the KEGG database (http://www.genome.jp/). The top 10 most enriched pathways were Intestinal immune network for IgA production, African trypanosomiasis, Inflammatory bowel disease, Transporters BR, Th1 and Th2 cell differentiation, Type 1 diabetes mellitus, Asthma, Cytokine receptors BR, Cytokine-cytokine receptor interaction, and Allograft rejection (Fig. 2). As described in the section of introduction, there is a close association between inflammatory and cognitive dysfunction in diabetes. Therefore, in the KEGG analysis, we focused on signaling pathways related to inflammation, with “Intestinal immune network for IgA production” ranking as the top pathway. Within this pathway, the level of IL 5 is significantly elevated in hippocampus of db/db mice, compared with db/m mice.

### GO enrichment analysis

**Fig. 3:**
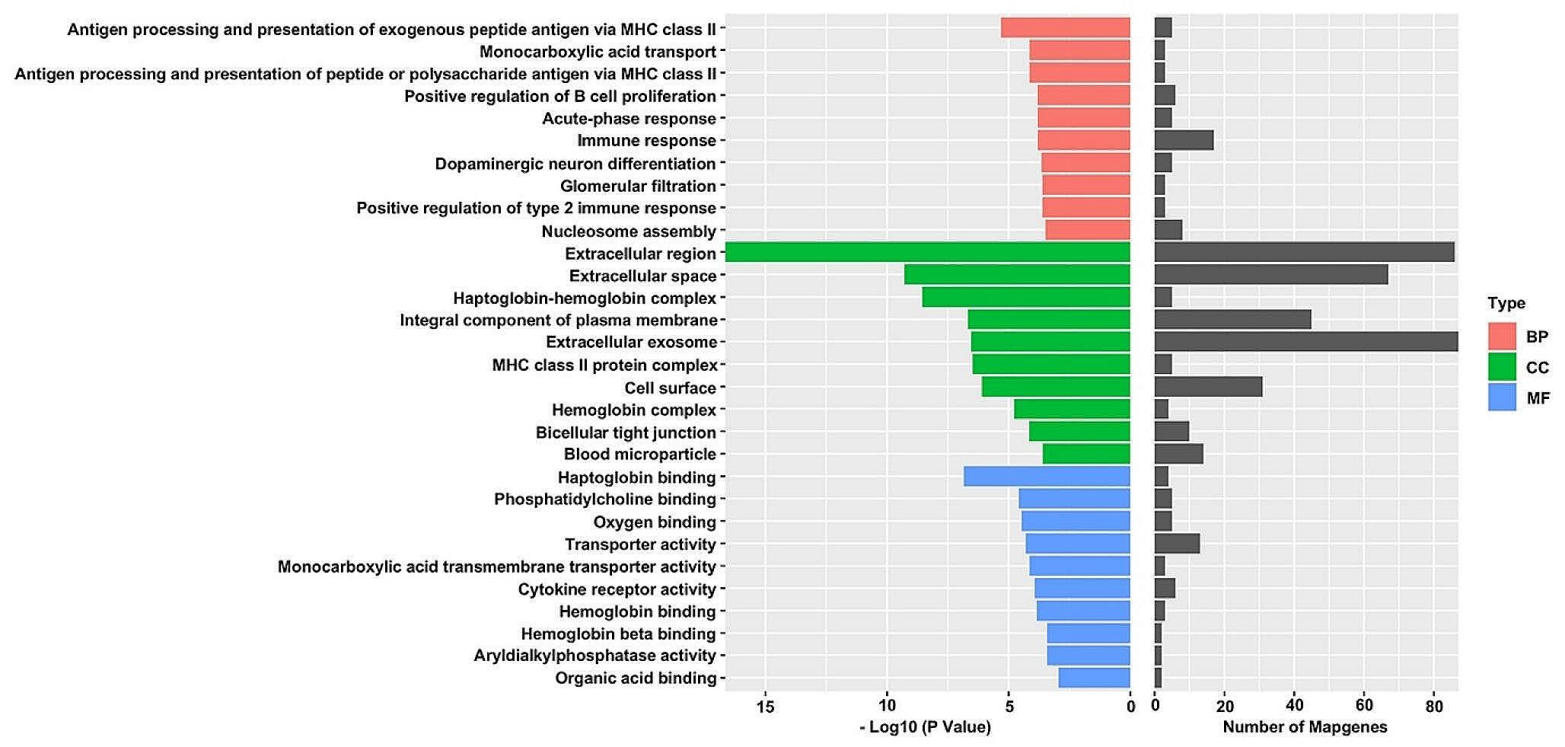
GO pathway analysis of DEGs in db/db mice and db/m mice. Notes for Fig. 3: The red bars represent the Biological Process (BP). The green bars represent the Cellular Component (CC) while the blue represent the Molecular Function (MF)

To interpret the possible biological roles of the DEGs in db/m mice and db/db mice, we made function annotation for each gene through GO functional enrichment analysis (Fig. 3). As for the biological process (BP), the genes were mainly enriched in Antigen processing and presentation of exogenous peptide antigen via MHC class II, Monocarboxylic acid transport, Antigen processing and presentation of peptide or polysaccharide antigen via MHC class II, Positive regulation of B cell proliferation, Acute-phase response, Immune response, Dopaminergic neuron differentiation, Glomerular filtration, Positive regulation of type 2 immune response, and Nucleosome assembly. Figure 3 lists the main GO terms that were enriched in the differentially regulated genes. Consequently, it was found that IL-5 involve in the top 1 KEGG pathway (Intestinal immune network for IgA production) and top 3 GO pathway (Antigen processing and presentation of peptide or polysaccharide antigen via MHC class II). So, the expression IL-5 was investigated in this present study.

### Validation of RNA-seq results

**Fig. 4:**
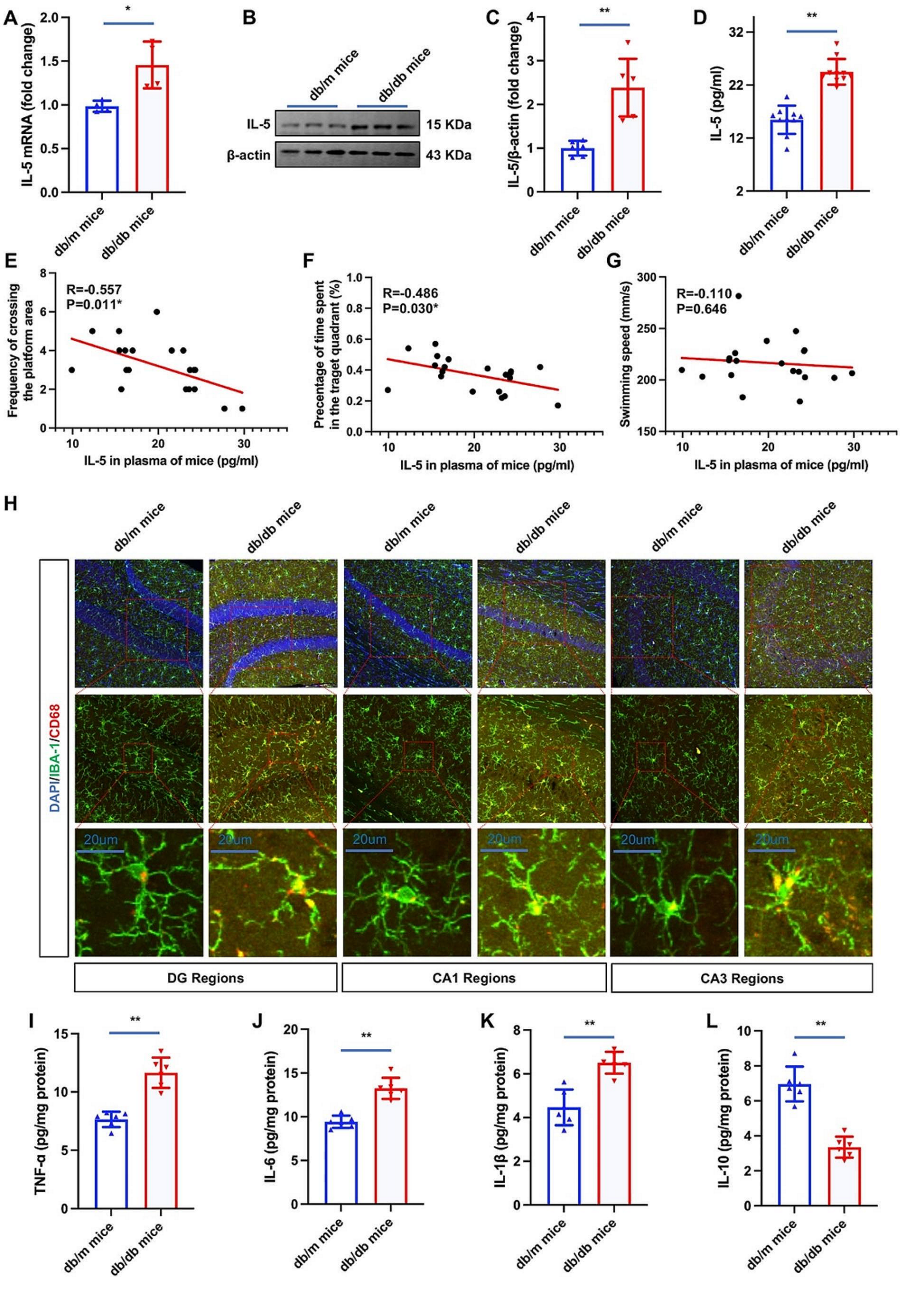
IL-5 levels and neuroinflammation of mice with and without diabetes. Notes for Fig. 4: ^*^P < 0.05, ^**^P < 0.01; Fig. 4A showed the IL-5 mRNA levels in db/db mice and db/m mice. Figure 4B showed the image of western blotting for IL-5, while Fig. 4C represent results of western blotting showed in Fig. 4B. Figure 4D showed levels of IL-5 in plasma of db/db mice and db/m mice. Figure 4E F showed the association between IL-5 levels in plasma and frequency of crossing the platform area (or percentage of time spent in the target quadrant) in mice. Figure 4G showed the correlation between IL-5 levels in plasma and swimming speed in mice. Figure 4 H showed the co-location of IBA-1 and CD68 in hippocampus of db/db mice and db/m mice. Figure 4I, 4 J, 4 K and 4 L showed TNF-ɑ, IL-6, IL-1β, and IL-10 levels in hippocampus of db/db mice and db/m mice

Real-time PCR was executed on cDNA samples to substantiate the expression profiles of IL-5 mRNA. Notably, our investigation revealed a marked increment in the levels of IL-5 mRNA within the hippocampal tissue of db/db mice in comparison to their db/m counterparts (Fig. 4A). Furthermore, a similar expression pattern was discerned in the protein levels of IL-5 (Fig. 4B and C), emanating from the expression of the IL-5 gene, mirroring the observed profile of IL-5 mRNA.

### Levels of IL-5 in plasma of mice without and with diabetes

In addition to the assessment of IL-5 levels within the hippocampus, quantification of protein levels in the plasma of mice was conducted. Analogous to the observed trend in the hippocampus, the IL-5 levels in the plasma of diabetic mice exhibited a statistically significant increase when compared to their nondiabetic counterparts (depicted in Fig. 3D). Moreover, noteworthy correlations were established between IL-5 levels in plasma and behavioral parameters, specifically the frequency of crossing the platform area (depicted in Fig. 3E) and the percentage of time spent in the target quadrant (Fig. 3F). While the statistical analysis did not reveal a significance in the association between IL-5 levels and swimming speed, it is pertinent to note that the overall profiles of these variables demonstrated a similar pattern (Fig. 3G).

### Neuroinflammation in hippocampus of mice without and with diabetes

The present study investigates the association of IL-5 with inflammation and its neuroprotective effects on the neuroinflammation within the hippocampus of murine subjects, with and without diabetes. Intriguingly, immunofluorescence analysis coupled with confocal microscopy revealed a heightened presence of CD68 co-localized with IBA-1 in the hippocampus of mice (Fig. 3H). Furthermore, quantitative assessment of isolated hippocampal protein demonstrated elevated levels of pro-inflammatory cytokines, namely TNF-ɑ (Fig. 3I), IL-6 (Fig. 3J), and IL-1β (Fig. 3K) concomitant with a reduction in the anti-inflammatory cytokine IL-10 (Fig. 3L). These findings underscore the putative involvement of IL-5 in modulating neuroinflammatory processes within the hippocampus, particularly in the diabetic condition.

### Comparison of clinical characteristics in T2DM patients with and without MCI

In this present study, we firstly analyzed the clinical data of recruited patients with T2DM. As a cross-sectional study, the age and gender of patients in MCI group differ from those of patients in control group. Our results showed that patients with MCI were significantly older than those with normal cognition (*P* = 0.004), and the percentage of females in the control group was significantly higher than in the MCI group (*P* = 0.010). However, all other relevant factors, including education levels, BMI, and levels of HbA1c, FPG, TG, TC, HDL-C, and LDL-C, were well-matched between the two groups (all *P* > 0.05), as shown in Table 1.

**Table 1:** Comparation of clinical parameters, cognitive function preference and IL-5 levels between control and MCI group in patients with T2DM

	Control (*n* = 70)	MCI (*n* = 35)	*P*
Age (year)	55.50 (49.00, 59.00)	59.00 (54.00, 65.00)	0.004^b*^
Female (n, %)	20, 28.57	19, 54.29	0.010^c*^
Education	12.00 (9.00, 15.00)	12.00 (6.00, 12.00)	0.118^b^
BMI (Kg/m^2^)	24.99 (22.25, 27.05)	24.24 (22.50, 25.98)	0.605^b^
HbA1c (%)	8.61 ± 1.85	8.70 ± 1.35	0.807^a^
FPG	7.58 (5.95, 10.02)	7.21 (5.82, 8.94)	0.373^b^
C peptide	517.97 (96.95, 692.04)	414.73 (292.12, 589.00)	0.210^b^
TG (mmol/l)	1.43 (1.00, 2.24)	1.49 (0.79, 2.36)	0.765^b^
TC (mmol/l)	3.96 (3.52, 4.94)	4.35 (3.33, 5.36)	0.552^b^
HDL-C (mmol/l)	0.97 (0.85, 1.16)	1.01 (0.90, 1.20)	0.386^b^
LDL-C (mmol/l)	2.72 ± 0.82	2.72 ± 0.75	0.965^a^
IL-5 (pg/ml)	22.21 ± 2.57	23.89 ± 1.87	0.001^a*^
MoCA	28.00 (27.00, 28.00)	23.00 (21.00, 25.00)	0.000^b*^
DST	11.00 (10.00, 13.00)	10.00 (9.00, 12.00)	0.009^b*^
VFT	18.00 (16.00, 21.00)	14.00 (12.00, 17.00)	0.000^b*^
TMTA	53.50 (42.50, 70.25)	75.00 (55.00, 102.00)	0.000^b*^
TMTB	137.50 (112.25, 175.00)	183.00 (143.00, 258.00)	0.000^b*^
LMT	9.00 (6.00, 13.25)	8.00 (4.00, 12.00)	0.096^b^

Notes:

a Student’s t test was employed for normally distributed variables

b The Mann-Whitney U test was employed for asymmetrically distributed variables

c The Chi-square test was employed for categorical variables

*P < 0.05

Abbreviations: IL-5, interleukin-5; MCI, mild cognitive impairment; BMI, body mass index; HbA1c, glycosylated hemoglobin; FPG, fasting plasma glucose; TG, triglycerides; TC, Total cholesterol; LDL-C, low density lipoprotein cholesterol; HDL-C, high density lipoprotein cholesterol; MoCA, Montreal Cognitive Assessment; DST, Digit Span Test; VFT, Verbal Fluency Test; TMTA, Trail Making Test-A; TMTB, Trail Making Test-B; LMT, Logical memory test

### Comparison of IL-5 levels and cognitive preference in T2DM patients with and without MCI

In this study, we aimed to explore the potential role of IL-5 in patients with T2DM who experience MCI. To achieve this objective, we compared the levels of IL-5 in plasma as well as the scores obtained from various cognitive tests, namely MoCA, DST, VFT, TMTA, TMTB, and LMT, between T2DM patients with and without MCI. We found that patients with impaired cognition had significantly elevated levels of IL-5 compared to those with normal cognition (*P* = 0.001). Furthermore, we observed that patients with MCI had decreased scores on MoCA, DST, and VFT, while increased scores on TMTA and TMTB, as compared to those without MCI (all *P* < 0.05). Although a decrease in LMT scores was also noted in diabetic patients with cognitive decline, compared to those with T2DM and normal cognition, there was no statistical significance (*P* = 0.096) (refer to Table 1 for detailed results).

### Association between IL-5 levels and cognitive preference in patients with T2DM

In order to investigate the potential correlation between IL-5 and cognitive function in patients with T2DM, Pearson association analyses were conducted. The results revealed interesting associations between IL-5 levels and MoCA scores (*R* = -0.389, *P* = 0.000; *R* = -0.349, *P* = 0.040), as well as TMTA scores (*R* = 0.192, *P* = 0.049; *R* = 0.047, *P* = 0.013), not only in all patients, but also in patients with MCI. Given the age and gender differences between patients with and without MCI, partial association analyses were also performed, which controlled for these two factors. Interestingly, the results showed that IL-5 levels were positively associated with TMTA scores in both all patients (*R* = 0.203, *P* = 0.039) and patients with MCI (*R* = 0.403, *P* = 0.020). Although IL-5 was not found to be significantly associated with MoCA scores in patients with MCI (*R* = -0.304, *P* = 0.086), it was significantly related to MoCA scores in all patients (*R* = -0.399, *P* = 0.000) (Table 2).

**Table 2:** Association between IL-5 and cognitive function in patients with T2DM

	Model 1				Model 2			
	All		MCI		All		MCI	
	R	P	R	P	R	P	R	P
MoCA	-0.389	0.000*	-0.349	0.040*	-0.399	0.000*	-0.304	0.086
DST	-0.110	0.266	-0.112	0.485	-0.092	0.357	-0.037	0.837
VFT	-0.044	0.657	-0.091	0.605	-0.037	0.710	-0.030	0.868
TMTA	0.192	0.049*	0.417	0.013*	0.203	0.039*	0.403	0.020*
TMTB	-0.182	0.063	0.179	0.302	0.204	0.039	0.173	0.337
LMT	-0.053	0.592	-0.239	0.167	-0.031	0.755	-0.166	0.356

Notes:

Model 1 showed the Pearson association between IL-5 and cognitive preference test scores in all patients and patients with MCI; Model 2 showed the partial association between IL-5 and cognitive preference test scores in all patients and patients with MCI adjusted for age and gender

*P < 0.05

Abbreviations: IL-5, interleukin-5; MCI, mild cognitive impairment; MoCA, Montreal Cognitive Assessment; DST, Digit Span Test; VFT, Verbal Fluency Test; TMTA, Trail Making Test-A; TMTB, Trail Making Test-B; LMT, Logical memory test

### Analysis for the risk factor for MCI and the factor influence MoCA and TMTA scores in patients with T2DM

In order to determine whether elevated levels of interleukin-5 (IL-5) serve as a risk factor for MCI in patients diagnosed with T2DM, binary logistic regression analysis was employed. The results indicate that heightened plasma concentrations of IL-5 are indeed a risk factor for MCI in T2DM patients (OR = 1.393, *P* = 0.002). Additionally, after adjusting for age and gender, elevated levels of IL-5 remained a risk factor for MCI in T2DM patients (OR = 1.472, *P* = 0.003) (Table 3). The present study conducted a multiple linear regression analysis to examine the relationship between IL-5 level and cognitive function as measured by the MoCA and the TMTA. The results indicated that IL-5 was a significant factor that influenced both MoCA score (*P* = 0.000) and TMTA score (*P* = 0.049) when age and gender were not adjusted for. After adjusting for age and gender, IL-5 remained a significant factor that impacted MoCA score (*P* = 0.000) and also affected TMTA score in patients withT2DM (*P* = 0.039) (Table 4).

**Table 3:** IL-5 is an independent risk factor for MCI in patients with T2DM

	β	OR	*P*
Model 1	0.331	1.393	0.002*
Model 2	0.386	1.472	0.003*

Notes:

Model 1 showed that IL-5 is a risk factor for MCI without the adjusting for age and gender; Model 2 showed that IL-5 is a risk factor for MCI adjusting for age and gender

*P < 0.05

Abbreviations: IL-5, interleukin-5; MCI, mild cognitive impairment

**Table 4:** Analysis for factors influence the TMTA scores of T2DM patients

		β	95% CL for β		P
			lower	upper	
MoCA	Model 1	-0.389	-0.749	-0.275	0.000*
	Model 2	-0.363	-0.694	-0.261	0.000*
TMTA	Model 1	0.192	0.009	5.081	0.049*
	Model 2	0.171	0.112	4.401	0.039*

Notes:

Model 1 indicated that IL-5 was a significant factor that influenced both MoCA score and TMTA score when age and gender were not adjusted for; Model 2 indicated that IL-5 was a significant factor that influenced both MoCA score and TMTA score after adjusting for age and gender

*P < 0.05

Abbreviations: TMTA, Trail Making Test-A; T2DM, type 2 diabetes mellitus; IL-5, interleukin-5

### Evaluation the diagnosis values of IL-5 for MCI in patients with T2DM

**Fig. 5:**
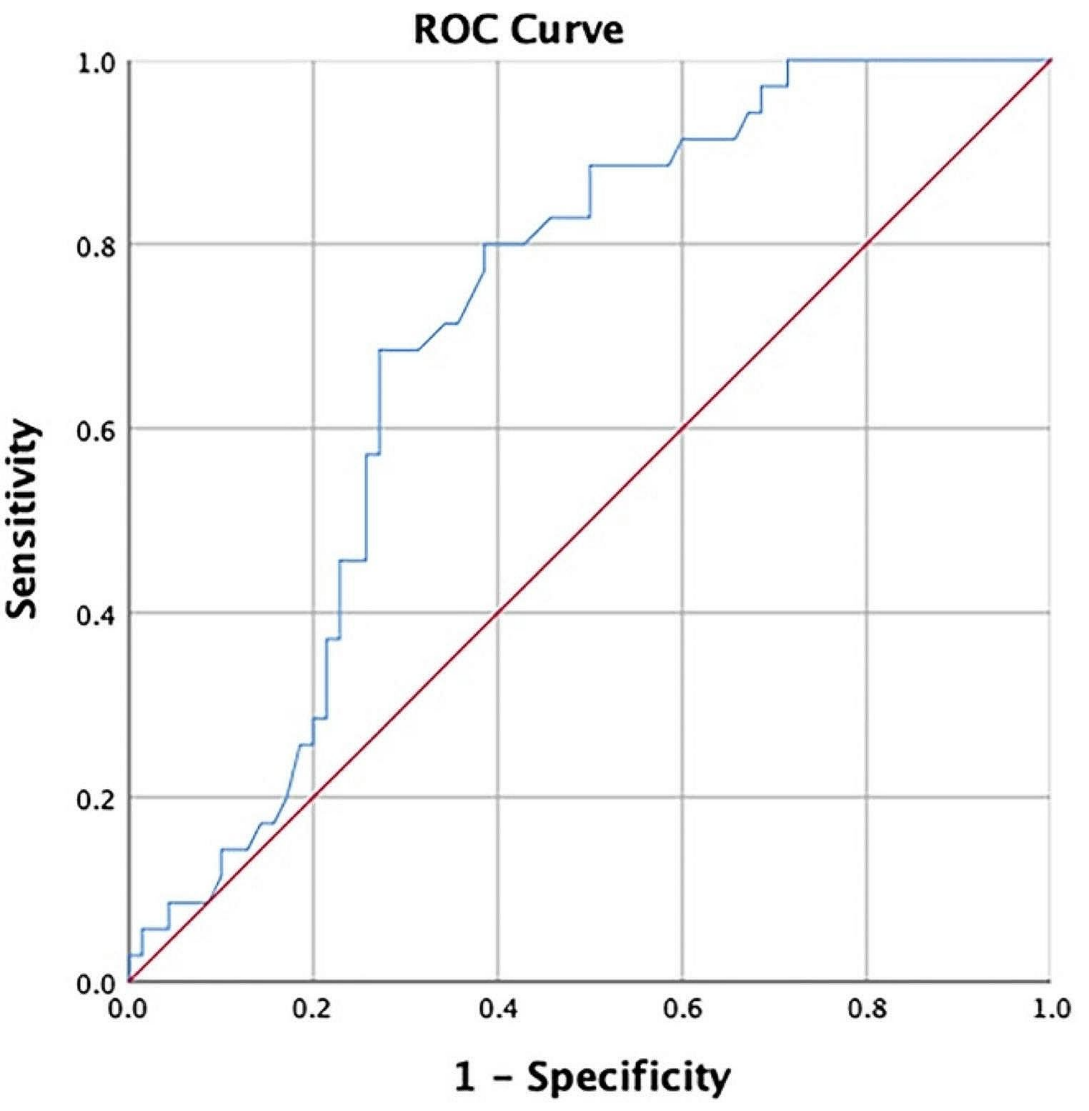
ROC curve of IL-5 for the sensitivity and specificity of MCI. Notes for Fig. 5: It is determined that the diagnostic cut-off value for IL-5 to be 22.98 pg/ml, revealed a sensitivity of 68.6% and specificity of 72.90%. The area under the curve is 0.711. The highest J point of Youden’s index is 0.415. Abbreviations: ROC, receiver operating characteristic; IL-5, interleukin-5; MCI, mild cognitive impairment

As an increased level of IL-5 has been identified as a potential risk factor for MCI in patients with T2DM, we conducted a detailed investigation to ascertain the diagnostic utility of IL-5. Using ROC curve analysis, we determined the diagnostic cut-off value for IL-5 to be 22.98 pg/ml (with the highest J point of Youden’s index: 0.415). Furthermore, our analysis revealed a sensitivity of 68.6% and specificity of 72.90% for the established cut-off value (Fig. 5).

## Discussion

The rising prevalence of diabetes has led to an increased focus on its complications, owing to their significant economic burden [47, 48]. MCI is one of the most important complications of diabetes, which if left untreated, can progress to dementia. However, this disease exhibits a hidden onset, mild symptoms, and lacks effective biomarkers, leading to delayed diagnosis [49]. The hippocampus is recognized as a crucial organ underlying cognitive functions. Numerous studies have demonstrated structural and functional impairments in the hippocampus of individuals suffering from cognitive dysfunction [50–52]. Our research also reveals that hippocampal tissues in diabetic mice with cognitive impairments exhibit excessive phosphorylation of Tau protein, a hallmark of cognitive dysfunction in diabetes [53].

**Fig. 6:**
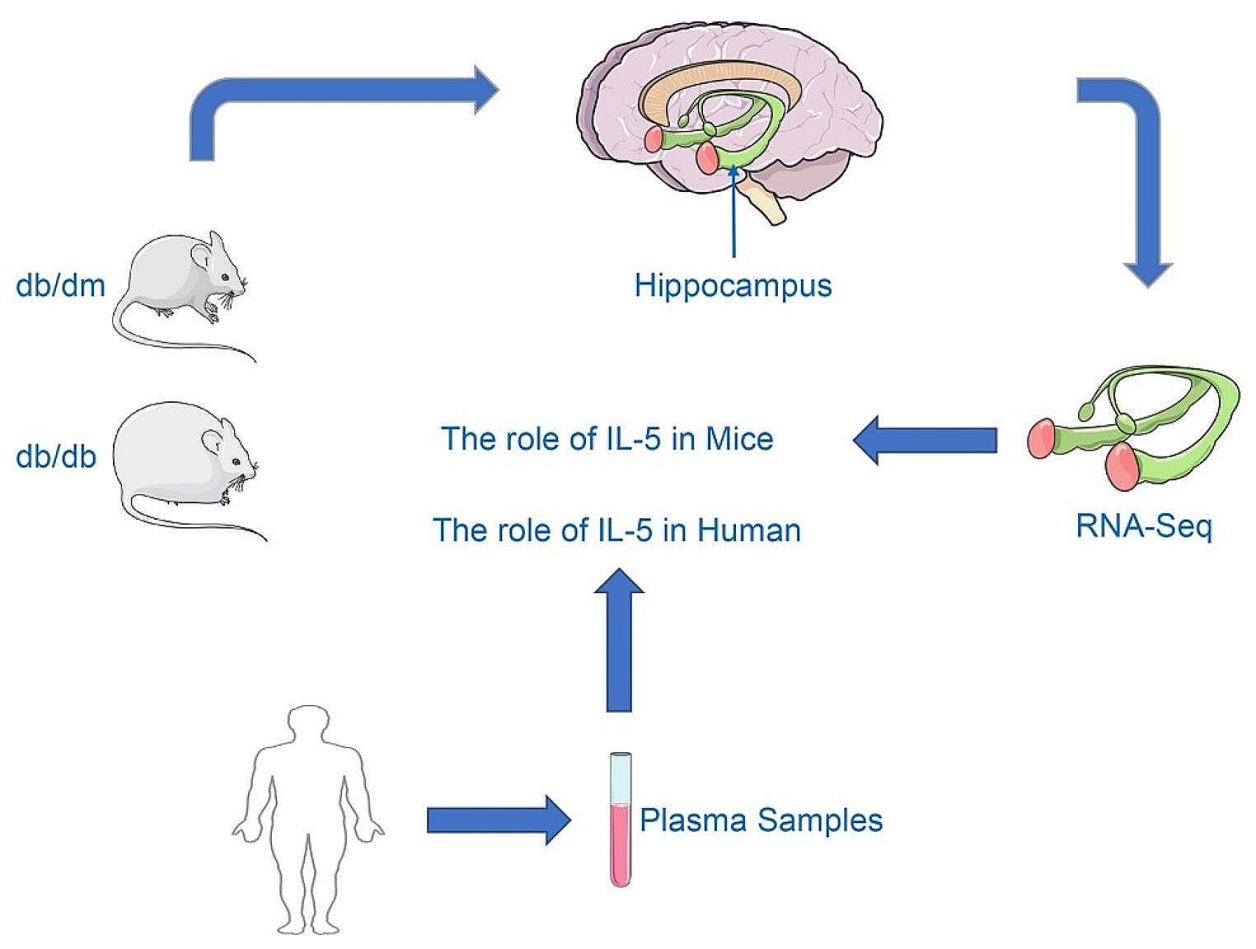
The main content of the research. Notes for Fig. 6: In this study, we first compared the RNA-seq results between diabetic mice (db/db mice) and control mice (db/dm mice). We then validated these sequencing results at both the mRNA and protein levels. Finally, we conducted clinical results to confirm the correlation between IL-5 and cognitive impairment and assessed the diagnostic value of IL-5 for MCI in patients with T2DM

In this study, we first compared the RNA-seq results of hippocampal tissues from diabetic mice and control mice. We then validated the sequencing results using PCR. Subsequently, we confirmed these results at the protein level. Through clinical experiments, we verified the correlation between IL-5 and cognitive impairment, and evaluated the diagnostic value of IL-5 for MCI in patients with T2DM (Fig. 6).

Firstly, in our present study, we initially employed transcriptome sequencing to elucidate the genes associated with changes in the hippocampal tissues of diabetic mice with cognitive dysfunction. As stated in the introduction, the emergence of diabetic MCI may stem from neuroinflammation [54]. Additionally, due to the significant involvement of inflammatory responses in cognitive impairment and metabolic disorders [55–57], particular attention was directed towards those associated with inflammatory responses. The results indicate that IL-5 is involved in the top-ranking KEGG pathway (Intestinal immune network for IgA production) and the third-ranking GO pathway (Antigen processing and presentation of peptide or polysaccharide antigen via MHC class II). Consequently, the expression of IL-5 was scrutinized in the current study. Further foundational research unveiled elevated levels of IL-5 not only in the hippocampal tissues but also in the plasma of diabetic mice. Additionally, the increased levels of IL-5 in the plasma demonstrated a correlation with the cognitive function of diabetic mice. Additionally, levels of IL-5 elevated in humans [21] and animals [58] with diabetes. Increased IL-5 levels have been linked to impaired cognitive function [17, 18] and may be associated with brain-derived neurotrophic factor [25], which plays an essential role in the development of diabetic cognitive impairment [59]. Thus, explore the potential links between IL-5 and cognitive function in patients with T2DM, and evaluate the diagnostic value of IL-5 for MCI in T2DM patients.

To the best of our knowledge, this is the first study to analyze the levels of IL-5 and cognitive dysfunction in patients with T2DM. In this present study, we firstly described the clinical characteristics of diabetic patients who were recruited. Although most factors were well-matched, there were differences in age and gender between diabetic participants with and without MCI. Therefore, these two factors were considered in further analysis. Secondly, we confirmed that IL-5 levels were increased in diabetic patients with MCI, which was consistent with studies demonstrating increased IL-5 levels in patients with other diabetic complications [60, 61]. Furthermore, we found that IL-5 levels were not only associated with the MoCA scores, which reflect global cognitive function, but also with the TMTA scores, reflecting information processing speed, in patients with T2DM. Moreover, these associations remained significant even after adjusting for age and gender in partial association analysis. These results are consistent with several other studies that have found associations between IL-5 and diabetic nephropathy and diabetic retinopathy [23, 48]. Additionally, IL-5 is a cytokine associated with inflammation and is released from group II innate lymphoid cells. Previously study indicated an association between lymphocyte and cognitive impairment by a meta-analysis of observational studies [62].

In order to validate the relationship between IL-5 and MCI in patients with T2DM, both binary logistic regression and multiple linear regression analyses were conducted. The results indicated that elevated levels of IL-5 are a risk factor for MCI in T2DM patients, with or without adjusting for age and gender. Furthermore, increased IL-5 levels were found to impact not only the overall cognitive function, but also the speed of information processing in patients. These findings are consistent with previous studies that have demonstrated the association between IL-5 levels and cognitive function [19, 20]. Additionally, the diagnostic values of IL-5 for MCI were calculated, aside from its relationship with cognitive function.

In the present study, we have discovered elevated levels of IL-5 in patients diagnosed with MCI. Our findings have demonstrated that the increased IL-5 expression serves as one of the risk factors for MCI in patients with T2DM. To our knowledge, this is the first study to establish a correlation between IL-5 and cognitive performance in T2DM patients. Our investigations have further indicated that the heightened levels of IL-5 can significantly affect the function of information processing speed. Furthermore, we have conducted the initial evaluation of the diagnostic value of IL-5 for MCI in patients.

In this study, we examined the correlation between IL-5 and cognitive dysfunction in diabetes and explored the potential diagnostic value of IL-5 in diabetic cognitive impairment. However, in the clinical research segment, our IL-5 levels were measured from peripheral blood, neither originating from brain tissue nor cerebrospinal fluid. This may diminish its diagnostic utility. Nevertheless, there may still exist a certain relationship between peripheral blood IL-5 levels and central nervous system IL-5 levels. Firstly, despite the presence of the blood-brain barrier, the central nervous system and peripheral tissues share similar environments; for instance, the levels of glucose in the nervous system are correlated with those in the periphery. Therefore, there may be a correlation in IL-5 levels produced by inflammatory cells in the central nervous system and peripheral organs. Additionally, studies have suggested that in diabetic conditions, the functionality of the blood-brain barrier may be compromised, allowing substances that typically cannot pass through the blood-brain barrier [63], such as IL-5, to traverse it. Certain specialized structures, including extracellular vesicles such as exosomes [64], might facilitate the transport of IL-5 across the blood-brain barrier.

To the best of our knowledge, this is the first study to explore the relationship between IL-5 and cognitive function in type 2 diabetes patients. In this present study, we not only found a correlation between IL-5 and cognitive function, but also suggest that elevated IL-5 mainly affects the information processing speed of diabetic patients. Furthermore, this study also assessed the value of IL-5 in diagnosing MCI in patients with T2DM for the first time. This research may hold potential value in early detection of cognitive impairment in diabetes. However, this study has several limitations that need to be considered. Firstly, it is a cross-sectional study with unmatched individuals in the control and MCI groups. Therefore, only a correlation between IL-5 and cognitive preference could be demonstrated, rather than a causal relationship. Although further analysis adjusted for age and gender, this limitation must be acknowledged. While IL-5 may serve as a potential biomarker that can be easily detected in peripheral blood, its sensitivity and specificity are only 68.6% and 72.90%, respectively, indicating that additional biomarkers for combined diagnosis are necessary. Secondly, since drug treatments, including diabetes drugs, may have a potential impact on cognitive function [65–67], the medication using of patients should be taken into account. However, among the enrolled patients, we only collected information on the medications they were using at the time of enrollment, without detailed records of dosage, formulation, and duration of medication use. Due to limitations in sample size, some medications were only used by a few patients. Therefore, conducting a detailed analysis of medications is evidently not feasible in our current study. This should be considered a limitation of this work. However, in another study conducted by our former colleagues, the potential impact of different medications on cognitive function was explored through network meta-analysis [68]. Thirdly, based on prior research showing the substantial influence of smoking and alcohol consumption on diabetic complications [69–71], we’ve omitted smokers and drinkers from our study. While exploring the impact of smoking and alcohol consumption on cognitive function is intriguing, we did not include patients engaging in these behaviors as it was not the focus of our study, which could be considered a limitation. Similarly, secondhand smoke may also potentially affect the nervous system [72], yet due to a lack of exploring strategy, our study did not address this issue, which can also be viewed as a limitation of our study. Lastly, mechanistically, the binding of IL-5 to its receptor recruit phosphorylation of JAK. The phosphorylation of JAK can further activate the JAK-STAT signaling pathway in a cascade manner. The JAK-STAT signaling pathway may be involved in cell apoptosis, including neurons [26–30]. Although our research has demonstrated the correlation between IL-5 and cognitive impairments in diabetes, the activation and involvement of the IL-5-associated JAK-STAT signaling pathway in neuronal apoptosis and its contribution to cognitive impairments in diabetes are still subject to further investigation.

## Conclusion

Generally, the evidence suggests that IL-5 is linked to cognitive impairment in individuals with T2DM. Elevated levels of IL-5 represent a potential risk factor for MCI in T2DM patients, specifically affecting information processing speed. The identified diagnostic threshold for IL-5 in relation to MCI is 22.98 pg/ml, and may hold promise as a biomarker for individuals with T2DM. While our study has explored the potential utility of IL-5 as a biomarker for MCI in patients with T2DM, further investigation through large-scale research endeavors is warranted to comprehensively assess its clinical applicability.

## Data Availability

All data in this manuscript have been submitted to The First Affiliated Hospital of USTC for records. All data are available on reasonable request from corresponding authors.
